# Analysis of Frontal Sinus Dimensions According to the Skeletal Malocclusion in German Teenagers

**DOI:** 10.1590/0103-644020245964

**Published:** 2024-12-06

**Authors:** Maria Beatriz Carvalho Ribeiro de Oliveira, Guido Artemio Marañón-Vásquez, Isabela Ribeiro Madalena, Eva Paddenberg-Schubert, Svenja Beisel-Memmert, César Penazzo Lepri, Ângela Graciela Deliga Schroder, Peter Proof, Christian Kirschneck, Erika Calvano Küchler, Maria Angélica Hueb de Meneze-Oliveira

**Affiliations:** 1 Department of Biomaterials, University of Uberaba, Uberaba, Minas Gerais, Brazil; 2 School of Dentistry of Ribeirão Preto, University of São Paulo, Ribeirão Preto, São Paulo, Brazil; 3 Department of Orthodontics, University of Regensburg, Regensburg, Germany; 4 Department of Orthodontics, University of Bonn, Bonn, Germany; 5 Graduate program, Tuiuti University of Paraná, Curitiba, Brazil

**Keywords:** Paranasal Sinuses, Frontal sinus, Malocclusion, Angle Class II, Malocclusion, Angle Class III, Adolescent

## Abstract

The present study explored the association between the craniofacial sagittal skeletal patterns and frontal sinus dimensions in Germans. This cross-sectional study included orthodontic patients with age ranging from 11 to 18 (80 males, 82 females). Lateral cephalograms were used in the cephalometric and frontal sinus analysis. Cephalometric analysis was performed to measure SNA, SNB and ANB angles. Frontal sinus measurements were performed to obtain the frontal sinus height, antero-posterior dimension and frontal sinus lateral projection area. The frontal sinus dimensions were compared between genders with Mann Withney test. Kruskal Wallis and Dunn's post-hoc tests were used to compare the mean differences between the skeletal malocclusions. Spearman’s correlation test and linear regression model were also performed. Statistical significance was set at 5%. Regarding skeletal malocclusion, 71 patients presented class I, 81 class II and 10 class III skeletal malocclusion. The frontal sinus height (p=0.009), frontal sinus antero-posterior dimension (p=0.001) and frontal sinus lateral projection area (p=0.007) were bigger in males than in females. The frontal sinus antero-posterior dimension was significantly greater in the class III when compared to the class I (p=0.010) and class II (p=0.027). Frontal sinus lateral projection area was also bigger in class III than class I (p=0.039). In the linear regression model a significant association was observed between frontal sinus lateral projection area and class III (skeletal class I as a reference). In conclusion, our result suggests that the frontal sinus lateral projection area might present morphometric differences in German teenagers with skeletal class III malocclusion.

## Resumo

O presente estudo teve como objetivo explorar a associação entre o padrão esquelético craniofacial sagital e a dimensão do seio frontal em adolescentes alemães. Tratou-se de um estudo transversal que incluiu pacientes ortodônticos alemães com idade variando entre 11 e 18 anos (80 homens, 82 mulheres). Cefalogramas laterais foram utilizados na análise cefalométrica e do seio frontal. A análise cefalométrica foi realizada para mensuração dos ângulos SNA, SNB e ANB. Medidas do seio frontal foram realizadas para obtenção da altura e dimensão ântero-posterior do seio frontal. A área da projeção lateral do seio frontal também foi determinada. As dimensões do seio frontal foram comparadas entre os sexos pelo teste de Mann Withney. Teste de Kruskal Wallis e pós teste de Dunn foram utilizados para comparar as diferenças entre as más oclusões esqueléticas. Também foram realizados o teste de correlação de Spearman e um modelo de regressão linear. A significância estatística foi estabelecida em 5%. Quanto à má oclusão esquelética, 71 pacientes apresentavam classe I, 81 classe II e 10 classe III. A altura (p = 0,009), dimensão ântero-posterior (p = 0,001) e área da projeção lateral do seio frontal (p = 0,007) foram maiores nos homens do que nas mulheres. A dimensão ântero-posterior do seio frontal foi significativamente maior na classe III quando comparada à classe I (p = 0,010) e classe II (p = 0,027). A área da projeção lateral do seio frontal também foi maior na classe III do que na classe I (p = 0,039). No modelo de regressão linear foi observada associação significativa apenas entre a área da projeção lateral do seio frontal e a classe III. Nossos resultados sugerem que a área da projeção lateral do seio frontal pode presentar diferenças morfométricas em adolescentes com maloclusão esquelética Classe III.

## Introduction

Frontal air sinus is a pneumatic cavity in the frontal bone and is one of the paranasal sinuses. It is part of the paranasal sinus respiratory system ^1^. The frontal sinus is directly related to the nasal cavity, and it is situated in the posterior part of the superciliary arches, between the external and internal faces of the frontal bone ^2^ ^,^ ^3^ ^,^ ^4^. Because of its highly variable anatomy and unique morphology, it is of interest in forensic medicine. The investigation of the frontal sinus morphology is useful in gender determination ^1^ ^,^ ^5^ ^,^ ^6^ and has been reported as a unique and reliable method to identify individuals ^3^ ^,^ ^7^.

Other research areas, in which the frontal sinus is analyzed, are craniofacial growth and development as well as orthodontics, and evidence can be found, that the growth and development of the frontal sinus are related to the development of different skeletal malocclusions (8- 16). According to the literature, the dimensions of the frontal sinus, which can be analyzed using lateral cephalograms, are associated with sagittal malocclusions ^11^ ^,^ ^12^ ^,^ ^13^ ^,^ ^14^ ^15^ ^,^ ^16^. Some authors also proposed that the frontal sinus could be used as an additional predictor for forecasting skeletal malocclusion of an individual ^17^. Skeletal class, which defines the relationship of the position of the upper and lower jaw in sagittal direction, can be divided into the three categories I, II and III, referring to a neutro-, disto- and mesio-sagittal jaw relation respectively. Since skeletal sagittal malocclusions, such as skeletal class II and III, are common craniofacial disorders, knowledge about the factors and aspects associated with these craniofacial disorders is important in different areas. Early prognostic factors regarding the development of malocclusions are an advantage in orthodontic therapy. Therefore, associations of such possible prognostic factors and skeletal malocclusions should be elucidated ^18^.

There is some evidence in the literature that frontal sinus morphology also varies according to the ethnic groups ^6^ ^,^ ^19^ ^,^ ^20^ and according to the age. It is known from previous studies, that the growth peak of the frontal sinus occurs at about 1 year after the growth peak of the body ^20^, making teenagers the group with the most developmental changes ^21^. Therefore, in the present study we aimed to explore the association between skeletal sagittal patterns and frontal sinus dimension in German teenagers.

## Materials and Methods

### The Study design

This manuscript is part of a larger research project that aims to study craniofacial development. The project was approved by the Human Ethics Committee of the University of Regensburg (number 19-1549-101). The study was conducted according to the ethical principles of Helsinki Declaration. Informed consent was obtained from all patients and their parents or legal guardians. Additionally, an assent document was used for patients younger than 14 years.

The Strengthening the Reporting of Observational Studies in Epidemiology (STROBE) recommendations, which consist of a checklist of 22 items ^22^, was used to design and report this study.

The recruitment and the collection for this cross-sectional study were previously described ^23^. This project included German orthodontic patients that were consecutively recruited during dental appointments from 2020 to 2021. For this study, the sample size was determined based on a previous study ^13^ with a power of 80% and a significance level of 5%. For the sample estimation, the mean differences and standard deviations among skeletal malocclusions observed in Gupta et al. ^13^ for height, antero-posterior dimension and frontal sinus lateral projection area were used. The sample size calculation estimated a size ranged from 6 to 12 per group. Due to the expected frequency of skeletal class III malocclusion in the target population, the sample size to be screened should be 180 patients.

### Sample description, inclusion and exclusion criteria

This study included only non-syndromic teenagers (11 to 18 years old). Patients with syndromes, adults (older than 18 years), children younger than 10 years old patients with congenital alterations including agenesis of permanent tooth/teeth (except for third molar agenesis), patients with cleft lip and/or palate, and patients with facial trauma and cases in which the frontal sinus could not be visualized were excluded. To minimize ethnicity variance and maximize the data interpretation, only patients with a self-reported Middle-European ancestry were included.

### Lateral cephalometric analysis for skeletal malocclusion and frontal sinus dimensions

Digital lateral cephalograms which were part of the patients’ orthodontic record, were used to perform the cephalometric analysis and the frontal sinus morphometric analyses.

The cephalometric measurements were performed by two trained and calibrated orthodontists that presented a good inter-examiner and intra-rater reliability as previously reported ^23^, briefly, the intra-class correlation coefficients (ICC) were used to calculate both, the intra and inter-rater reliability. Inter-examiner reliability ranged from 0.95 to 0.98, while the intra-examiner reliability ranged from 0.91 to 0.97.

**Figure 1:**
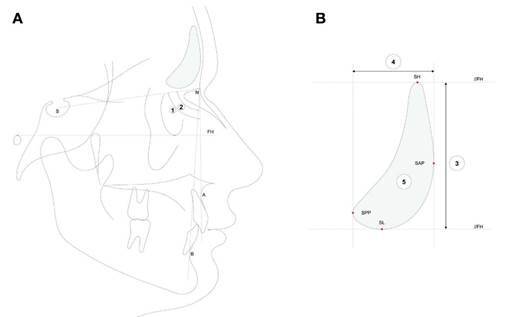
Cephalometric and frontal sinus measurements evaluated. A- Cephalometric landmarks: S - Sella, N - Nasion, A - point A; B - point B, angle 1 = SNB (sagittal position of the mandible), angle 2 = ANB (skeletal class - intermaxillary sagittal relationship). B- Frontal sinus landmarks: SH - most superior point, SL - most inferior point, SAP - most anterior point, SPP - most posterior point, 3 - height of the frontal sinus, 4 - frontal sinus antero-posterior dimension, 5 - Frontal sinus lateral projection area. FH means Frankfurt Horizontal plane.

The radiographs were performed in different sets, but followed the same protocol, in which the patients were instructed to keep their teeth in habitual occlusion, lips relaxed and be comfortable in the upright position, with the eyes focused on a point in a distance at eye level. All the radiographs were taken using cephalostat and Frankfurt Horizontal plane parallel to floor. Every radiographic setting used the exposure settings according to the manufactures recommendation. All lateral cephalometric radiographs were imported as lossless TIF files into the software ivoris analyze pro (Computer konkret AG, Falkenstein, Germany, version 8.2.15.110) and calibrated. Cephalometric analysis was performed digitally based on Segner and Hasund ^24^. The anatomical landmarks point A, point B, Sella (S), and Nasion (N) were determined manually using the cephalometric analysis software ivoris^®^ analyze pro (Computer konkret AG, Falkenstein, Germany, version 8.2.15.110), and the angular measurements SNA (Sella - Nasion - A point angle), SNB (Sella - Nasion - B point angle) and ANB (A point- Nasion- B point angle) were calculated using cephalometric analysis software. All the angles and landmarks used are demonstrated in the Figure 1. The sagittal skeletal malocclusions were defined based ion the ANB, as follows:

- All skeletal Class I malocclusion patients had an ANB angle value between 0° and 4°,
- All skeletal Class II malocclusion patients had an amplitude of ANB angle value >4°,
- All skeletal Class III malocclusion patients had an amplitude of ANB angle value <0°.

Frontal sinus measurements were performed by a trained and calibrated dentist with 5 years of experience who had good intra-examiner reliability, with an ICC ranging from 0.91 to 0.94. The radiographs were initially positioned with the Frankfurt horizontal plane (lowest point of the below edge of the orbits and the upper edge of the ear-aperture) parallel to the floor. Using Adobe Photoshop CS6 (Adobe Systems Inc., San José, CA, USA), two horizontal reference lines, parallel to Frankfurt horizontal plane, passing through the most superior (SH) and most inferior (SL) points of the frontal sinus were created. Likewise, two vertical reference lines, perpendicular to the Frankfurt horizontal plane, passing through the most anterior (SAP) and most posterior (SPP) points, were drawn. Edited radiographs were imported into the Image J software (NIH, Bethesda, MD, USA), where their size was calibrated based on the ruler included in the images. Using the 'straight' tool, the height (perpendicular distance between both horizontal reference lines) and antero-posterior dimension (perpendicular distance between both vertical reference lines) of the frontal sinus were measured. The frontal sinus lateral projection area was determined using the 'freehand selections' tool. Each measurement was performed in triplicate, and the mean value was used for subsequent analyses. The landmarks and measurements evaluated are presented in Figure 1.

### Statistical analysis

To confirm that the data were normally distributed, the Shapiro-Wilk test was used. The frontal sinus dimensions were compared between genders with Mann Withney test. Kruskal Wallis and Dunn's post-hoc test for multiple comparisons were used to compare the mean differences of the frontal sinus dimensions between the three types of skeletal malocclusions. The correlation between cephalometric angular dimensions and the frontal sinus dimensions was evaluated using Spearman’s correlation test. To control for the influence of age and gender in the frontal sinus dimensions and skeletal malocclusions, a multivariate linear regression model was performed to investigate if the association observed in the Kruskal Wallis with Dunn's post-hoc remained.

All statistical analyses were carried out on GraphPad Software version 9 for Mac (GraphPad Software, San Diego, CA, USA). Statistical significance was defined as an alpha of 5% (p < 0.05).

## Results

A total of 187 patients were screened, 1 patient was excluded due to cleft lip and palate, 13 patients older than 18 years old were excluded, 8 patients had tooth agenesis and for 3 patients it was not possible to visualize the frontal sinus. Finally, 162 patients (80 males and 82 females) were included in this study. Regarding the skeletal malocclusions, 71 patients presented skeletal malocclusion I (neutral jaw configuration), 81 patients presented skeletal malocclusion II and 10 patients presented skeletal malocclusion III.

**Figure 2:**
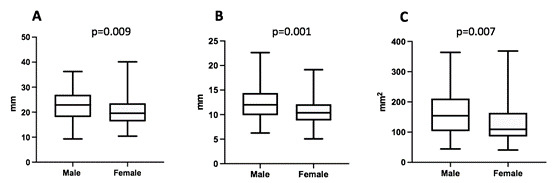
Frontal sinus dimensions according to gender. A - Frontal sinus height; B - Frontal sinus antero-posterior dimension; and C - Frontal sinus lateral projection area.

The dimensional characteristics of the included sample is presented in table 1. Figure2 shows the frontal sinus dimensions according to gender. Frontal sinus height (Figure 2A) ranged from 9.30 to 36.25 in males (median = 29.89), while in females ranged from 10.42 to 40.14 mm (median = 20.54), a statistical significance was observed (p = 0.009). The frontal sinus antero-posterior dimension (Figure 2B) ranged from 6.24 to 22.62 mm in males (median = 12.01), while in females raged from 5.06 to 19.15 mm (median = 10.37), a statistical significance was observed (p = 0.001). Frontal sinus lateral projection area (Figure 2C) ranged from 44.32 to 364.0 mm^2^ in males (median = 154.1), while in females raged from 40.71 to 368.3 mm^2^ (median = 109.3), a statistical significance was observed (p=0.007).

**Table 1:** Measurements of the studied population

Dimensions	Minimum	Maximum	Median
ANB(°)	-5.20	9.30	4.05
SNA(°)	75.0	93.2	81.05
SNB(°)	66.0	90.2	76.90
Frontal sinus height (mm)	9.30	40.15	20.64
Frontal sinus antero-posterior dimension (mm)	5.06	22.62	11.19
Frontal sinus lateral projection area (mm^2^)	40.71	368.30	132.3

Mann Whitney test was used.

**Figure 3:**
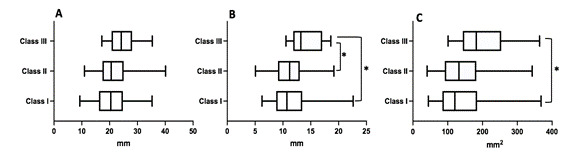
Frontal sinus dimensions according to the skeletal malocclusions. A - Frontal sinus height; B - Frontal sinus antero-posterior dimension; and C - Frontal sinus lateral projection area. *Means statistically significant difference. Kruskal Wallis and Dunn's post-hoc tests were used.

Figure 3 shows the frontal sinus dimensions according to the skeletal malocclusions. The frontal sinus height (Figure 3A) ranged from 9.30 to 36.26 (median = 20.43) in skeletal class I, from 10.96 to 40.14 (median = 21.04) in skeletal class II, and from 17.22 to 35.36 (median = 24.18) in skeletal class III. A statistical significance was not observed (p > 0.05). The frontal sinus antero-posterior dimension (Figure 3B) ranged from 6.24 to 22.62 (median = 10.71) in skeletal class I, from 5.06 to 19.15 (median = 11.20) in skeletal class II, and from 10.55 to 18.06 (median = 13.21) in skeletal class III. A statistical significance was observed between skeletal class I and skeletal class III (p = 0.010) and between skeletal class II and skeletal class III (p = 0.027). Frontal sinus lateral projection area (Figure 3C) ranged from 44.32 to 368.31 (median = 120.6) in skeletal class I, raged from 40.71 to 342.8 (median = 132.30) in skeletal class II, and ranged from 101.10 to 364.0 (median = 182.0) in skeletal class III. A statistical significance was observed between skeletal class I and skeletal class III (p = 0.039).

**Figure 4:**
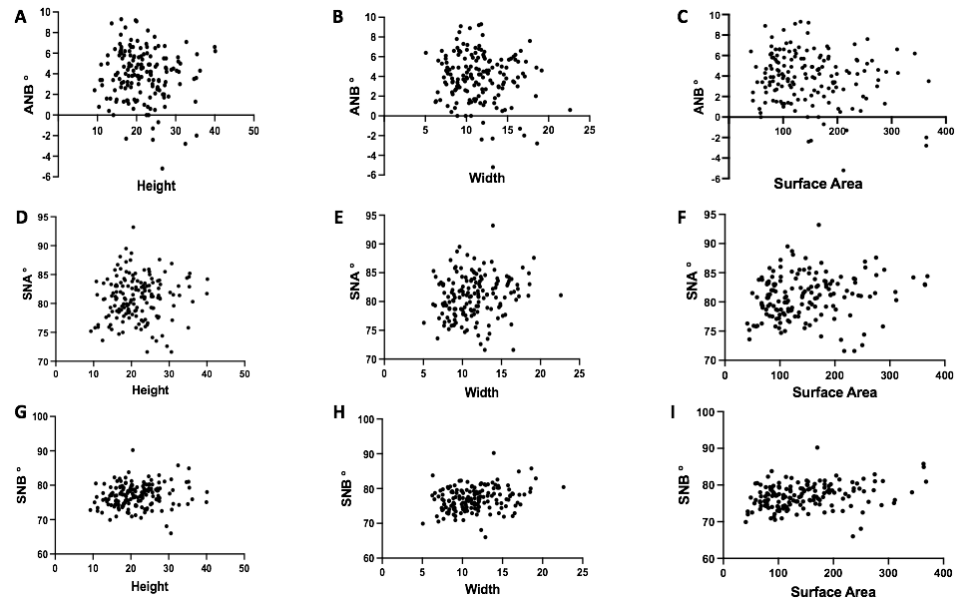
Correlation between the frontal sinus dimensions and cephalometric indices. A- Correlation between ANB and height (r=-0.073); B- Correlation between ANB and frontal sinus antero-posterior dimension (r=0.128); C- Correlation between ANB and frontal sinus lateral projection area (r=0.152); D- Correlation between SNA and height (r=0.085); E- Correlation between SNA and frontal sinus antero-posterior dimension (r=0.156); F- Correlation between SNA and frontal sinus lateral projection area (r=0.160); G- Correlation between SNB and height (r=0.147); H- Correlation between SNB and antero-posterior dimension (r=0.262); and I- Correlation between SNB and frontal sinus lateral projection area (r=0.160). Spearman’s correlation test was used.

The Spearman’s correlation analysis of the frontal sinus height dimension, antero-posterior dimension, and frontal sinus lateral projection area with the cephalometric indices are presented in Figure 4. The results showed a statistically significant association between frontal sinus lateral projection area and skeletal class III malocclusion (skeletal class I as a reference). The associated results are presented in Table 2.

**Table 2:** Multivariate linear regression model adjusted for age and gender

	Variable	Estimate	p-value
Frontal sinus lateral projection area	Skeletal malocclusion class III	50.62	0.030*
Frontal sinus antero-posterior dimension	Skeletal malocclusion class II	-0.30	542
	Skeletal malocclusion class III	1.31	200

Note: Comparison reference was skeletal class I. * means statistically significant difference.

## Discussion

Skeletal malocclusions are the most prevalent developmental anomalies of craniofacial structure with a life-long morbidity and have been associated with several conditions as well as pointed as causative factor for other disorders ^18^. It has been suggested that more studies investigating possible factors associated with skeletal malocclusions are required to increase diagnostic precision and to optimize treatment options ^18^. Although skeletal malocclusions, which result from disharmonious positions of the maxilla and/ or mandible, can occur in the sagittal, frontal and transverse direction, we focused on sagittal discrepancies in our study, since they were previously described to show an association with frontal sinus dimensions in other populations ^11^ ^,^ ^12^ ^,^ ^13^ ^,^ ^15^ ^,^ ^16^.

Lateral cephalograms present a standard diagnostic tool in orthodontics to evaluate sagittal malocclusions, which refer to the antero-posterior imbalances between the maxilla, mandible and/ or other craniofacial structures. To assess the skeletal relationship between the maxilla, the mandible among each other and to the cranial base, the angles SNA, SNB and ANB were used as parameters describing the degree of prognathism of the upper and lower jaw and the skeletal class, respectively. Although the ANB angle has some limitations, such as the disregard of its dependence on other craniofacial structures, it is often used in treatment planning as well as in orthodontic and craniofacial research ^25^. Furthermore, lateral cephalograms were used in our study to investigate frontal sinus dimensions. Even though this method has been commonly used in recent studies ^8^ ^,^ ^9^ ^,^ ^10^ ^,^ ^11^ ^,^ ^12^ ^,^ ^13^ ^,^ ^15^, there are important limitations that should be highlighted here. The inherent limitation of conventional two-dimensional radiographic images for measuring complex three-dimensional anatomical structures is a limitation of our study, specially due the lack of volume analysis. Although a computed tomography scan would allow a more accurate analysis of the frontal sinus dimensions on both sides, this retrospective study was based on the records of non-syndromic orthodontic patients available, i.e., on their lateral cephalograms. Since three-dimensional radiographic imaging is not routinely used in orthodontic diagnostics and treatment planning, the data available and the retrospective study-design did not allow a three-dimensional analysis of the frontal sinus dimensions.

In our study, skeletal malocclusion was associated with frontal sinus antero-posterior dimension and frontal sinus lateral projection area, and skeletal class III patients presented higher dimensions. Similar results were previously reported. Sabharwal et al. ^11^ observed that the antero-posterior dimension and frontal sinus lateral projection area were statistically significantly greater in Indian class III patients. Tunca et al. ^16^ observed that the frontal sinus height and antero-posterior dimension were bigger in Turkish patients with small ANB angles. Yassaei et al. ^12^ also reported that dimensions and frontal sinus lateral projection area were statistically significantly greater in Iranian class III patients. Following the same pattern, Algahefi et al. ^15^ investigated Caucasian and Chinese samples and observed that the frontal sinus area was also greater in class III patients. Gupta et al. ^13^ reported that the frontal sinus lateral projection area and antero-posterior dimension were statistically significantly larger in Class III and smaller in Class II patients. In our sample, however, skeletal class II and I patients presented similar measurements.

The frontal sinus morphology and dimensions vary among different ethnicities ^6^. Although previous studies suggested the association between skeletal malocclusions and frontal sinus dimensions, none of them investigated European samples. Therefore, in this study we could show that our results followed a similar pattern as other populations worldwide. Another important aspect, which needs to be considered when comparing our findings with other studies, is that our data rely on adolescent patients. Since the frontal sinus growth starts at the age of two years, reaches its peak approximately one year after the pubertal growth spurt ^20^ and continues until approximately 18 years ^12^, our findings may be affected by this possible confounder. Another factor, which is associated with frontal sinus dimension, is gender. A recent systematic review with meta-analysis evaluated 18 studies and concluded that the frontal sinus are dimorphic structures and potential anatomical landmarks useful for the sex estimation process in forensic ^5^. Gender difference was also observed in our sample for all evaluated measurements. Hence, a linear regression analysis was performed using gender and age as variables in this analysis, and skeletal class III still was associated with frontal sinus lateral projection area.

According to our findings, the angular measurements ANB, SNA and SNB do not show a correlation to the frontal sinus measurements. This could be possibly explained by the small number of patients with a negative ANB, i.e. skeletal class III. In fact, the prevalence of classes I and II are much higher in comparison to class III ^18^. Although our sample size calculation predicted that this sample would be large enough to detect mean difference, future studies should include larger samples of skeletal class III patients to investigate the correlation of the continuous variables. Furthermore, future study design should also investigate patients at different time periods or after finishing growth, to consider growth as a possible confounder. Thereby, the precision of the finding that frontal sinus could act as a potential predictor for skeletal class III and the need for orthognathic surgery ^16^.

## Conclusion

Our result suggests that the frontal sinus lateral projection area might present morphometric differences in German teenagers with skeletal class III malocclusion. This finding may be useful in the orthodontic early diagnostics and treatment planning in the future.
